# Recyclable Graphene Sheets as a Growth Template for Crystalline ZnO Nanowires

**DOI:** 10.3390/nano11082093

**Published:** 2021-08-18

**Authors:** Yeonhoo Kim, Dongheun Kim, Eric Auchter, Justin Marquez, Roxanne Tutchton, Nan Li, Ting S. Luk, Enkeleda Dervishi, Yong-Jin Kim, Jian-Xin Zhu, Jinkyoung Yoo

**Affiliations:** 1 Center for Integrated Nanotechnologies, Los Alamos National Laboratory, Los Alamos, NM 87545, USA; yeonhoo@lanl.gov (Y.K.); heun82@gmail.com (D.K.); elauchter@gmail.com (E.A.); jmarquezche@gmail.com (J.M.); nanli@lanl.gov (N.L.); 2 T-4, Los Alamos National Laboratory, Los Alamos, NM 87545, USA; rtutchton@lanl.gov (R.T.); jxzhu@lanl.gov (J.-X.Z.); 3 Center for Integrated Nanotechnologies, Sandia National Laboratories, Albuquerque, NM 87185, USA; tsluk@sandia.gov; 4 SIGMA-2, Los Alamos National Laboratory, Los Alamos, NM 87545, USA; 5 School of Physics and Astronomy, University of Manchester, Oxford Road, Manchester M13 9PL, UK; dibykim@gmail.com

**Keywords:** graphene, ZnO, recycling, hydrothermal synthesis, raman spectroscopy

## Abstract

Recent advances in nanoscience have opened ways of recycling substrates for nanomaterial growth. Novel materials, such as atomically thin materials, are highly desirable for the recycling substrates. In this work, we report recycling of monolayer graphene as a growth template for synthesis of single crystalline ZnO nanowires. Selective nucleation of ZnO nanowires on graphene was elucidated by scanning electron microscopy and density functional theory calculation. Growth and subsequent separation of ZnO nanowires was repeated up to seven times on the same monolayer graphene film. Raman analyses were also performed to investigate the quality of graphene structure along the recycling processes. The chemical robustness of graphene enables the repetitive ZnO nanowire growth without noticeable degradation of the graphene quality. This work presents a route for graphene as a multifunctional growth template for diverse nanomaterials such as nanocrystals, aligned nanowires, other two-dimensional materials, and semiconductor thin films.

## 1. Introduction

Epitaxy of thin films and nanomaterials is a crucial step for device manufacturing. However, conventional epitaxy is preferably conducted when original substrates and overgrown materials possess similar lattice constants and thermal expansion coefficients. To utilize the epilayer, the substrates are mostly disposed of or used as a base substrate in devices. Since the cost of substrates for epitaxy process takes up a significant portion in device manufacturing, there has been an intensive research effort to recycle substrates. Epitaxial lift-off (ELO) is a technique of recycling wafers [1], a typical process sequence for ELO is formation of sacrificial layer, epitaxy of desired materials, and post-processing to release the epitaxial thin films and nanomaterials. Although the ELO technique has demonstrated the reuse of substrates for various device applications, such as thin film photovoltaic cells, photodetectors, and light-emitting diodes [1,2,3], it still requires chemo-mechanical polishing, which induces substrate material loss of ~10 µm/recycle. The material loss results in a process cost increase comparable to ~20% of the substrate price [4].

Graphene provides novel opportunities for substrate recycling because of its self-terminated surface without surface dangling bonds, which usually act as origins of interfacial defects and strong chemical bonding between graphene and an overgrown material. Successful demonstrations of van der Waals epitaxy and remote epitaxy show that graphene is a versatile substrate for epitaxial growth of elemental and compound semiconductors [5,6,7,8,9,10,11]. ELO on graphene can protect the substrate underneath this 2D graphitic structure. However, in this case, the graphene film cannot be recycled because it delaminates from the substrate or it starts forming micro-spalling marks. As of now, large-scale production of high-quality graphene at a low cost is still challenging, given that the use of graphene as a sacrificial layer for the ELO process could lead in a manufacturing cost increase [12]. Although graphene has shown a great potential as a growth template for nanomaterials with optimized morphologies and characteristics, recyclability of graphene nanosheets for repetitive nanomaterial growth has rarely been investigated [13]. Furthermore, ZnO nanostructures have been studied extensively due to their stable and uniform growth on graphene for various potential applications, such as biomedical and optoelectronic devices [14,15]. The growth mechanism of ZnO nanostructures has also been explored to investigate the influence of the nature of graphene layers [16].

In this work, we report a recycling process of monolayer graphene used as a growth template for synthesis of single crystalline ZnO nanowires (NWs). The single crystalline ZnO NWs were selectively grown on the graphene. Moreover, the selective nucleation of ZnO on graphene was investigated by first-principles calculations based on density functional theory (DFT). Hydrothermal synthesis and delamination processes using chemical exfoliation of the ZnO wires were repeated on a same graphene layer. Raman spectroscopy analysis demonstrates that the graphene layer remains stable after multiple NW synthesis and delamination processes. In addition, Raman mapping analysis was performed to investigate the quality of graphene surfaces along repetition of ZnO NW growth and subsequent delamination. The chemical robustness of graphene enables the reuse of the graphene layer for repeated nanomaterials growth (at least seven times) without noticeable degradation of its quality. This work provides a route for graphene as a recyclable growth template for various materials.

## 2. Experimental Details

Monolayer graphene was synthesized on a 0.1 mm thick Cu foil (CAS# 7440-50-8, purchased from Alfa Aesar) using a low-pressure chemical vapor deposition which was previously reported [17]. The Cu foil was annealed at 950 °C for 1 h under Ar/H_2_ atmosphere. Next, methane (CH_4_, 15 sccm) are introduced in the system while the temperature was increased to 1020 °C for 20 min. Lastly, the sample was rapidly cooled down to room temperature. The synthesized graphene sheet with a surface area of 1.5 cm × 1.5 cm was transferred onto a SiO_2_/Si substrate using a PMMA-free transfer method [18]. The transfer technique utilizes formvar (polyvinyl formal), a chemically stable and easy to remove polymer, for a rapid, clean and reliable transfer of large-area graphene sheets over a desired substrate.

E-beam lithography (EBL) was employed to define the nucleation sites of ZnO NWs on the graphene sheet. A 300 nm-thick poly (methyl methacrylate) (PMMA, MicroChem^®^ 495 K C3, Newton, MA, USA) layer was deposited by spin coating. The EBL was performed using the JEOL 6300 FS instrument (JEOL Ltd, Akishima, Tokyo, Japan). The *e*-beam exposed PMMA regions defined as dot patterns were removed by development in methyl isobutyl ketone (MIBK):isopropyl alcohol (IPA) solution. The remaining PMMA layer acted as mask of selective growth of ZnO NWs on the exposed graphene.

ZnO NWs were grown using hydrothermal method on the graphene/SiO_2_/Si substrate. The monolayer graphene/SiO_2_/Si template was placed upside-down in a Teflon-lined autoclave with the nutrient solution. The nutrient solution was prepared by dissolving zinc nitrate hexahydrate (25 mM, ZnO(NO_3_)_2_·6H_2_O), hexamethylenetetramine (25 mM, C_6_H_12_N_4_, HMTA), and polyethylenimine (5 mM, PEI) in deionized water. Zinc nitrate hexahydrate and HMTA were employed as precursors to form ZnO. PEI was used to induce growth along one principal direction by adsorbing onto nonpolar sidewalls of ZnO crystal. The synthesis was performed at 95 °C for 4 h. Details of the hydrothermal growth procedure are reported elsewhere [19].

Raman analyses for point spectra and mapping were taken by a homebuilt system, which was calibrated using a Si wafer. A frequency doubled 532 nm Nd:YAG laser was used to probe the nanostructures and the Raman signal was collected via a liquid nitrogen cooled CCD detector (Princeton Instruments, Inc., Trenton, NJ, USA). For the micro-photoluminescence (PL), a frequency quadrupled Nd:YAG laser with the wavelength of 266 nm, a pulse width of 400 ps, and a repetition rate of 10 kHz was employed as the excitation source.

## 3. Results and Discussions

**Figure 1:**
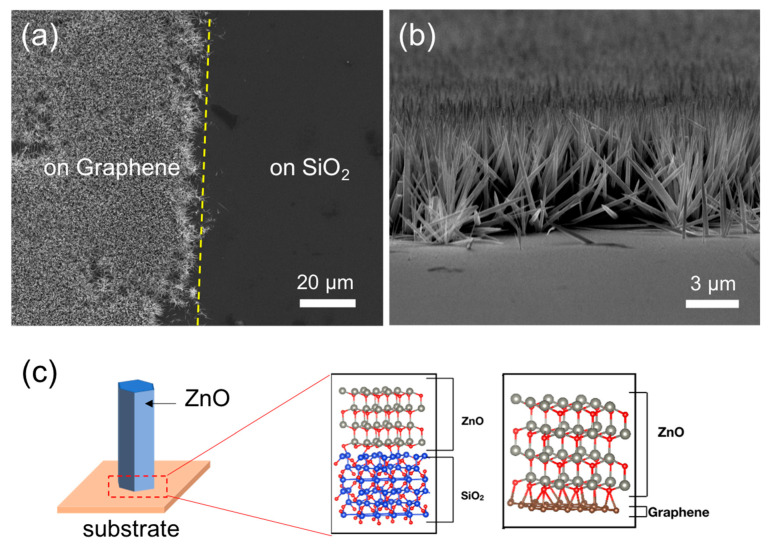
(**a**) Top-view and (**b**) tilted-view SEM images of ZnO wires grown on graphene. (**c**) The supercells composed of ZnO, SiO_2_, and graphene for the DFT calculations.

The surface morphology and the microstructures of the ZnO NWs were investigated by scanning electron microscopy (SEM, FEI Quanta 400 F, 10 kV, FEI Company, Hillsboro, OR, USA) and transmission electron microscopy (TEM, FEI Tecnai F20, 200 kV, FEI Company, Hillsboro, OR, USA). Figure 1a shows a selective growth of the ZnO NWs on graphene, while Figure 1b presents a tilted-view SEM image of ZnO NWs grown on graphene. The ZnO NWs were preferably grown on graphene, while no growth was observed on the SiO_2_ surface. Position-controlled hydrothermal growth of ZnO micro/nanostructures on graphene has been reported by several research groups [20,21,22,23]. Earlier, ZnO seed layers were employed to promote the growth of ZnO structures. However, direct growth of ZnO NWs on graphene without any seed layer has not been fully understood. Nucleation of ZnO crystallites by hydrothermal growth is explained by either homogeneous nucleation in a nutrient solution or heterogeneous nucleation on hydrophilic surfaces [19]. Our observation of selective nucleation of ZnO on graphene layer is incomprehensible by the conventional interpretations, since pristine graphene is not hydrophilic without undergoing additional chemical treatments for introduction of surface functional groups. To elucidate on the observation, the selective nucleation of ZnO on graphene was investigated by DFT calculations. Figure 1c shows the model systems for the DFT calculations. The super cells consisting of the ZnO structure bonded to commensurate supercells of SiO_2_ or graphene were formed to reduce required computational resource and approximate the binding energies between ZnO and the substrates without consideration of edge-effects. The total energies of supercells of ZnO, SiO_2_, and graphene were calculated based on DFT as implemented in the Vienna Ab-initio Simulation Package (VASP). All calculations were conducted with projector augmented-wave (PAW) potentials using the generalized gradient approximation (GGA) of Perdew, Burke, and Ernzerhof (PBE). Each model system was relaxed along the z-direction using preconditioned residual-minimization until the force on the atoms was less than 0.01 eV/Å. In these calculations, no edge or vacuum effects were considered.

The relative binding energy per Zn atom is defined as

(1) $$E_{B}=\left(E_{\mathrm{substrate}}+E_{\mathrm{ZnO}}-E_{\mathrm{ZnO}/\mathrm{substrate}}\right)/n,$$

where *E*_substrate_ is the total energy of the substrate, SiO_2_ or Graphene, *E*_ZnO_ is the total energy of the ZnO nanorod, *E*_ZnO/substrate_ is the total energy of the combined nanorod/substrate system, and *n* is number of Zn or O atoms at the surface of the substrate. The binding energies were calculated to be 0.3654 and 1.7864 eV for the ZnO/SiO_2_ and ZnO/graphene structures, respectively. The higher binding energy for ZnO/graphene compared to ZnO/SiO_2_ indicates that the growth of ZnO nanostructures is energetically more favorable on graphene than on SiO_2_ [24].

**Figure 2:**
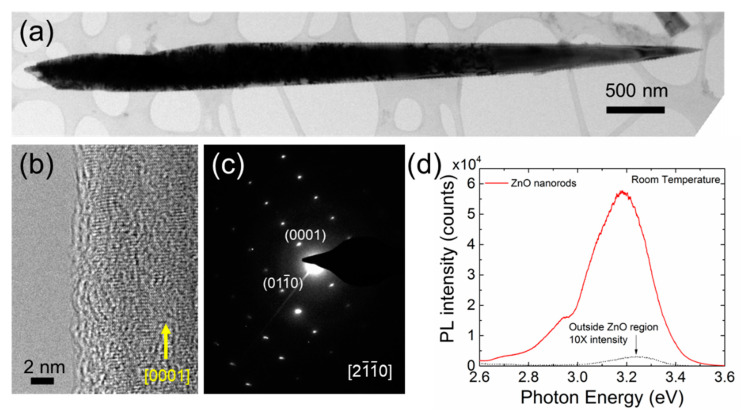
(**a**) Low magnification, (**b**) high-resolution TEM images, and (**c**) electron diffraction pattern of an individual ZnO NW grown on CVD-graphene via hydrothermal method. (**d**) Room temperature µ–PL spectra of ZnO NWs and SiO_2_ outside graphene.

TEM was used to investigate the structural characteristics of the ZnO NWs on graphene. Figure 2a shows a low magnification TEM image of a ZnO NW with a sharp tip. The tapered shape with sharp tips is commonly observed for ZnO NWs prepared by hydrothermal method under alkaline conditions and non-catalyzed growth via metal-organic CVD [5,15]. Figure 2b,c indicate that the *c*-oriented ZnO NW is single crystalline. The formation mechanism of sharp tips of the ZnO NWs can be attributed to surface energy anisotropy that (0001) *c*-plane has the highest surface energy in wurtzite ZnO. Hence, reduction of the surface area of (0001) plane is energetically favorable [25].

The optical properties of the ZnO NWs were investigated using micro-photoluminescence (µ–PL) spectroscopy at room temperature (RT). The strong PL emission centered at 3.25 eV on the graphene, shown in Figure 2d, corresponds to the free excitonic emission of ZnO [26]. Moreover, there was no noticeable emission in visible wavelengths, of which origins are corresponding to oxygen vacancies and zinc interstitials [27]. The luminescence from the SiO_2_ region was negligible. High crystallinity and dominant free excitonic emission in RT µ–PL spectrum of the ZnO NWs demonstrate that graphene can be employed as a growth template for high-quality ZnO nanostructures.

**Figure 3:**
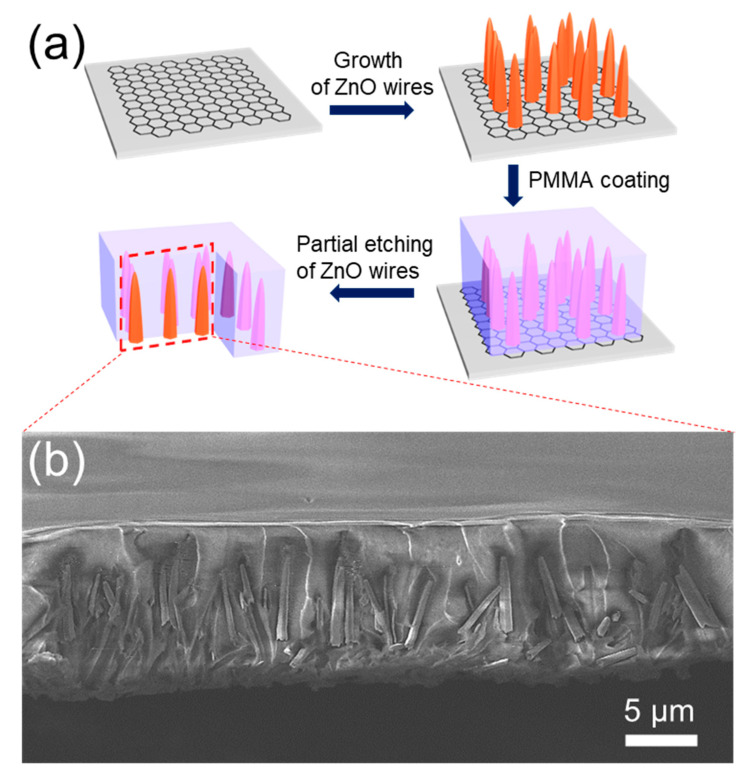
(**a**) A schematic of the process to separate ZnO wires from CVD-graphene by PMMA coating and partial wet etching processes. (**b**) Tilted-view SEM image of ZnO wires embedded in PMMA after separation from graphene.

To recycle graphene layers and repeatedly synthesize ZnO NWs, repetitive detachment processes of ZnO NWs are required. Mechanical exfoliation is an alternative way to separate the semiconducting materials from graphene surface. However, the mechanical exfoliation method easily damages either ZnO or graphene layer. Hence, we developed a wet chemical delamination process to simultaneously preserve the original structure of the ZnO NWs and that of the graphene layer. The slanted ZnO NWs (Figure 1b) result in an areal density gradient of the projected NWs region. While the roots of ZnO NWs are dense, the tips of the NWs are sparse. The ZnO NWs grown on the graphene layer were covered with PMMA via spin coating. PMMA solution (PMMA 950 K) was dropped on the ZnO NWs grown on graphene and baked at 180 °C on a hot plate for 3 min. To cover the tips of the NWs, the PMMA coating process was repeated three times. Because the radius of gyration of PMMA 950 K is 26 nm, the PMMA solution cannot permeate into a space narrower than the radius of gyration of 26 nm [28]. Hence, the bottom parts of the ZnO NW bundles were not covered with PMMA even after repetition of the PMMA coating. The PMMA/ZnO/graphene sample was immersed in 0.01 M hydrochloric (HCl) acid solution for 24 h to etch only the bottom parts of the ZnO NWs where PMMA was not covering. Figure 3a shows the PMMA coating and subsequent PARTIAL etching process of the ZnO NWs grown on the graphene layer. Figure 3b shows the separated ZnO NWs coated with the PMMA after the partial etching of ZnO with HCl. As the HCl solution at this particular molarity does not introduce any significant damage on graphene, SiO_2_, and Si, the repetitive growth and etch process can be successfully utilized to recycle the growth templates.

## 4. Raman Spectroscopy Analyses

**Figure 4:**
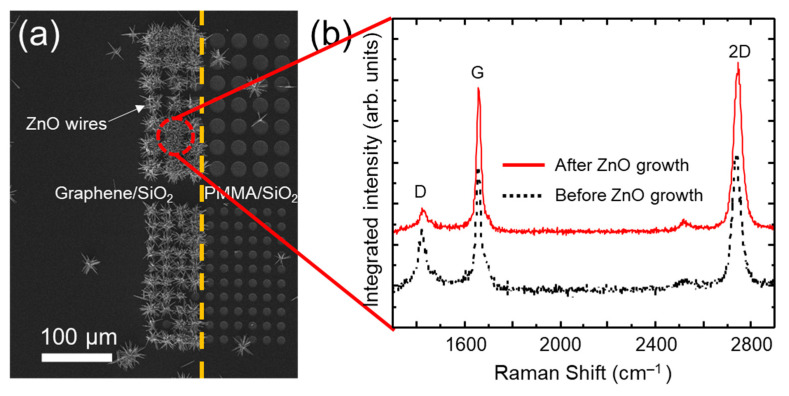
Top-view SEM (**a**) image of hydrothermally grown ZnO NWs on a hole patterned monolayer graphene/SiO_2_/Si substrate. The left and the right sides of the orange dashed line are the regions on monolayer graphene/SiO_2_/Si and SiO_2_/Si. (**b**) The room temperature Raman spectra of the monolayer graphene, obtained at the ‘red’ circle in (**a**), before (black, dashed) and after (red, solid) growth of ZnO NWs.

Raman spectroscopy is a non-destructive technique commonly used to probe various carbon nanostructures [29,30,31]. The position and intensity ratios of the characteristic Raman bands (D, G, and 2D band) of graphene are used to characterize crystallinity, number of layers, doping level, presence of defects and the type of functional groups, etc. A frequency doubled 532 nm Nd:YAG laser was employed to generate the Raman scattering on the graphene sheets before and after the removal of ZnO NWs via the wet etching. In order to characterize the same position on the graphene surface, the locations of ZnO NWs on graphene were defined by *e*-beam lithography. The graphene area was patterned to check the quality of the graphene layer on a defined spot during repetition of the cycle consisting of growth and separation of ZnO NWs. Figure 4a shows the SEM image of hydrothermally grown ZnO NWs on a hole patterned PMMA/monolayer graphene/SiO_2_/Si substrate. The observation of selective growth of ZnO NWs on the exposed graphene areas is consistent with the result shown in Figure 1. The area marked with the ‘Red’ circle in Figure 4b indicates the position where Raman spectra of graphene were acquired before and after growth of ZnO NWs. Raman spectra were also acquired after growth of ZnO NWs and before the partial etching of ZnO NWs. Figure 4b shows three distinct peaks at ~1420 (D), ~1650 (G), and ~2740 (2D) cm^−1^, which can be assigned to an A_1g_ breathing mode originating from disorders, to the high frequency E_2g_, and to the second order of the D, respectively [32]. Raman analyses were employed to monitor band intensities and ratios, hence providing information on graphene structural integrity. Raman spectroscopy is also a well-defined method to measure the number of graphene layers [32,33]. The Raman spectrum of the transferred graphene before the ZnO growth indicates the presence of a crystalline sheet [29]. To maintain the quality of the graphene during transfer, we used our newly developed PMMA-free transfer method while minimizing introduction of defects and avoiding impurities [18,34].

The Raman spectra shown in Figure 4b of the same graphene area before and after ZnO NWs growth exhibits similar spectral features. Across a series of point scans there was no change in the relative intensities of the D, G, and 2D peaks, nor was there a consistent shift in peak positions. Additionally, regions of graphene (with and without NWs) probed over a series of 50 scans did not show any significant difference in their corresponding Raman spectra (Figure S1). This was also confirmed by a detailed Raman mapping analysis of these characteristic bands collected in the graphene regions before and after NWs growth (Figure S2). These observations imply that the hydrothermal growth of ZnO NWs induce neither noticeable incorporation of new disorders in graphene nor alteration of chemical characteristics of graphene. The ZnO NWs cause some scattering of the beam, but their Raman spectral features are not within the key spectral range of graphene [35,36].

**Figure 5:**
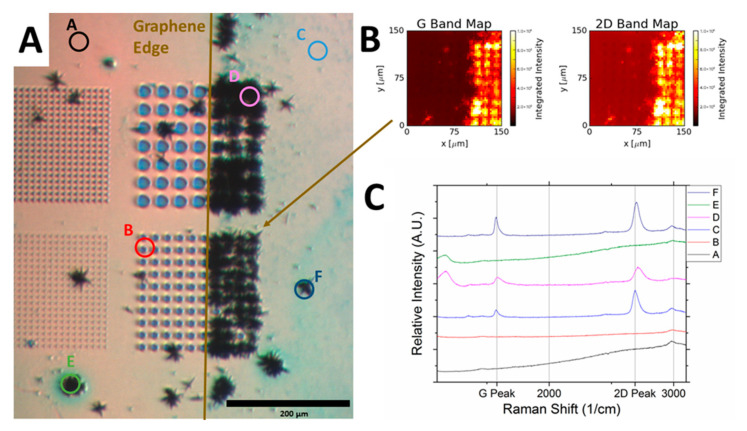
(**A**) Top-view optical microscopy image of a ZnO NWs/graphene sample with partial coverage of graphene. A–F indicate the locations where Raman measurements were done. (**B**) Raman intensity maps of G and 2D bands confirming graphene areas corresponding to the region of ZnO NWs growth. (**C**) Raman spectra collected at the points of A–F.

Raman mapping clearly reveals that ZnO NWs were grown on graphene as shown in Figure 5. Figure 5 exhibits a growth boundary for the NWs, which can be seen along the edge of graphene that bisects the patterned region. Raman intensity mappings (G and 2D band spectral maps) in Figure 5B show the strong signal response from the graphene where ZnO NWs were grown. The point spectra in Figure 5C indicate the graphene presence in the growth regions and the lack of it in other areas.

**Figure 6:**
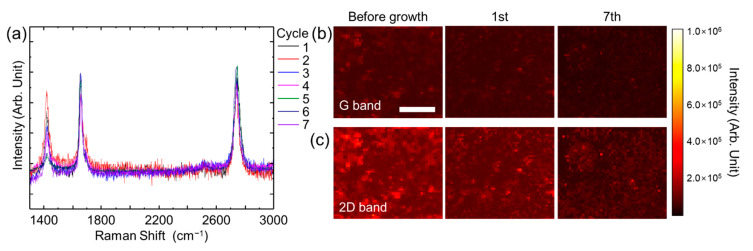
(**a**) Raman spectra of graphene layer over 7 times of repeated hydrothermal growth and removal of ZnO NWs. (**b**) Raman map of G and (**c**) 2D bands before the growth of ZnO NWs and after repetitive growth of ZnO NWs up to 7 times.

A key feature of recycling graphene for semiconducting nanomaterial growth is the remarkable properties that this 2D material continues to possess without degradation when repetitively used as a substrate. Thorough Raman spectroscopy analyses including point spectra and Raman mapping were performed over the growth area to characterize the quality of graphene along the repetition of ZnO NWs growth and subsequent delamination. The process of growth and delamination of ZnO NWs was repeated additional seven times on the same graphene. The overlays of all eight spectra of the graphene after each recycling process are shown in Figure 6a. The slight peak shift of ±15 cm^−1^ and no change in the relative intensities of the three major bands (D, G, 2D) were observed. The slight shift in values of ±15 cm^−1^ is likely due to environmental or equipment differences on the collection days instead of changes to the graphene structure [37]. Raman shifts have been reported to be affected by numerous environmental factors such as temperature, laser power, and excitation energy [38,39].

To further characterize the graphene structure after each growth and removal of nanowires, Raman mapping in a graphene area of 150 µm^2^ was conducted. Raman maps of G, and 2D bands in Figure 6b,c indicate no changes in any of the three band intensities after recycling the graphene seven times. The characterization was performed in other areas of graphene, probed after repeated hydrothermal growth and wet etching of ZnO NWs, yielding similar results (Figure S3). The graphene sheet was not deteriorated even after repeated growth and etching cycles of ZnO NWs over its surface, making it an attractive substrate for semiconducting nanomaterial growth.

**Figure 7:**
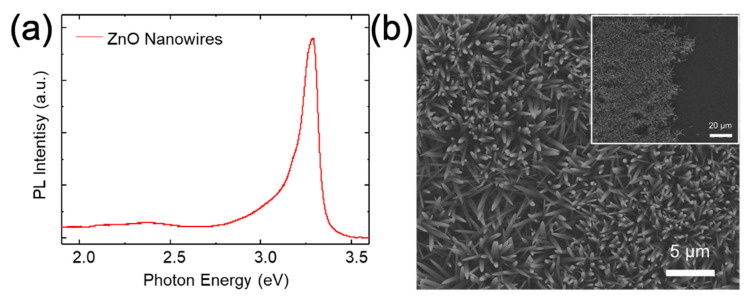
(**a**) RT PL spectrum of ZnO wires after the 4th cycle of the growth-etching process. (**b**) SEM images of ZnO wires grown on graphene after the 7th growth and etching cycle. The inset is a low magnification image of the ZnO wires. There was no growth of ZnO wires on SiO_2_ layer.

Structural and optical properties of the ZnO NWs grown at the different cycles of the growth-etching process were also characterized. Figure 7a shows the RTPL spectrum of ZnO NWs after the 4th cycle of the recycling process. The weak emission in visible wavelengths indicates low concentration of unintentional defects, such as oxygen vacancies and zinc interstitials. Figure 7b exhibits that the ZnO NWs maintained the identical morphology with same diameter and length after the 7th cycle of the recycling. In addition, ZnO NWs were not grown on SiO_2_ layer, as shown in the inset of Figure 7b, after repetition of the recycling process.

No noticeable degradation of materials quality in both recycled graphene and ZnO wires region shows that the recycling graphene process in this study is applicable to production of nanomaterials without concern of process-dependent material degradation.

## 5. Conclusions

Recycling of graphene as a growth template for single crystalline ZnO NWs was demonstrated by hydrothermal synthesis and chemical wet etching processes. The crystallinity and position-controlled growth of ZnO NWs were investigated by the SEM, and TEM analyses and interpreted by theoretical calculations. Raman analyses demonstrate that the graphene template was very stable and reliable after the repetitive growth and detachment processes. Our developed strategy for template-assisted synthesis of nanomaterials based on CVD graphene and recycling of the templates opens up a path forward for this graphitic material as an exciting growth substrate not only the research field, but also manufacturing and industrial fields.

## Data Availability

The data that support the findings of this study are available within this article and its Supplementary Materials.

## Supplementary Materials

The following are available online at https://www.mdpi.com/article/10.3390/nano11082093/s1. Additional PL and Raman spectra, Raman mapping data, SEM images are presented in Supplementary Materials.
