# Supplementary material for: Recyclable Graphene Sheets as a Growth Template for Crystalline ZnO Nanowires
Source: Nanomaterials (Basel). 2021 Aug 18;11(8):2093. doi: 10.3390/nano11082093 (PMC8399327; doi:10.3390/nano11082093)
Supplement: Supplementary file 1 [file nanomaterials-11-02093-s001.zip › nanomaterials-1302132-supplementary.pdf]

nanomaterials

Supplementary Materials

Recyclable Graphene Sheets as a Growth Template for Crystalline ZnO Nanowires

Yeonhoo Kim [1,†], Dongheun Kim [1,†], Eric Auchter [1], Justin Marquez [1], Roxanne Tutchton [2], Nan Li [1], Ting S. Luk [3], Enkeleda Dervishi [4,*], Yong-Jin Kim [5], Jian-Xin Zhu [2] and Jinkyoung Yoo [1,*]

1 Center for Integrated Nanotechnologies, Los Alamos National Laboratory, Los Alamos, NM 87545, USA; yeonhoo@lanl.gov (Y.K.); heun82@gmail.com (D.K.); elauchter@gmail.com (E.A.); jmarquezche@gmail.com (J.M.); nanli@lanl.gov (N.L.)
2 T-4, Los Alamos National Laboratory, Los Alamos, NM 87545, USA; rtutchton@lanl.gov (R.T.); jxzhu@lanl.gov (J.-X.Z.)
3 Center for Integrated Nanotechnologies, Sandia National Laboratories, Albuquerque, NM 87185, USA; tsluk@sandia.gov
4 SIGMA-2, Los Alamos National Laboratory, Los Alamos, NM 87545, USA
5 School of Physics and Astronomy, University of Manchester, Oxford Road, Manchester, M13 9PL, UK; dibykim@gmail.com
* Correspondence: enkeleda@lanl.gov (E.D.); jyoo@lanl.gov (J.Y.)
† These authors contributed equally to this work.

Raman spectra of graphene areas with and without nanowires appeared to be similar, as shown in Figure S**1.** The nanowire growth (regions with the yellow circle in Figure S1) did not induce any significant changes to the spectral shape or the G to D band intensity ratio. This might indicate that in terms of atomic structure of graphene as perceivable by Raman spectroscopy, has no clear effect on growth areas nor does the nanowire growth alter the graphene's morphological properties.

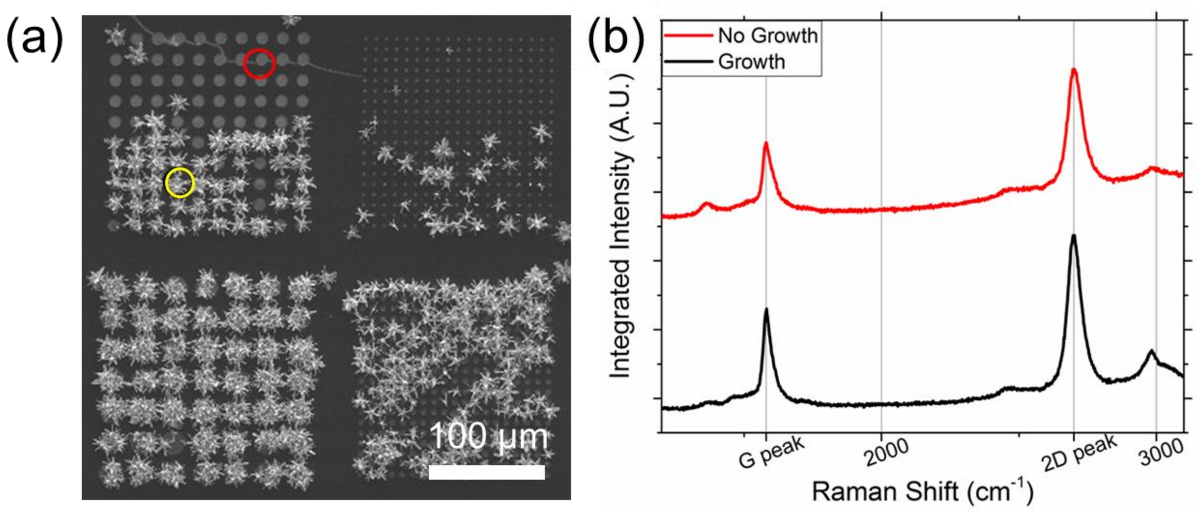

Figure S1. (**a**) SEM image of nanowire growth pattern with and without nanowires indicating regions for Raman spectroscopy. The red circle corresponds to the "No Growth" spectrum and the black circle corresponds to the "Growth" spectrum. (**b**) Raman spectra of graphene regions indicated by the circles in A.

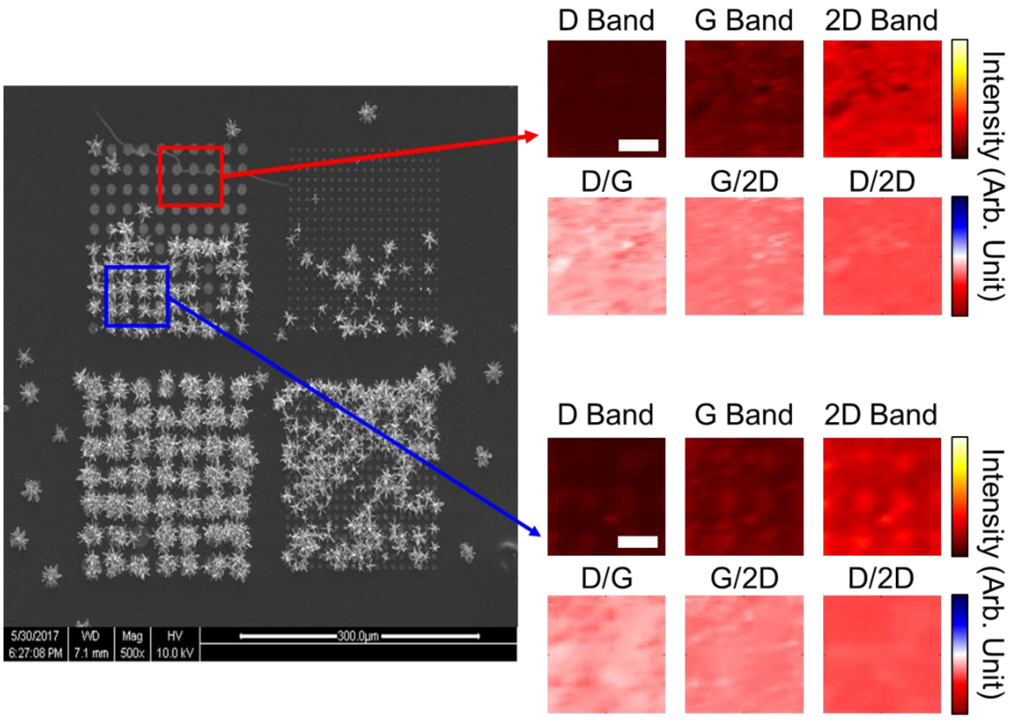

Figure S2. SEM image of nanowire growth pattern with and without nanowires indicating regions for Raman mappings. The red square corresponds to the Raman mapping (upper) of the graphene without nanowires and the blue square corresponds to the Raman mapping (lower) of the graphene with nanowires.

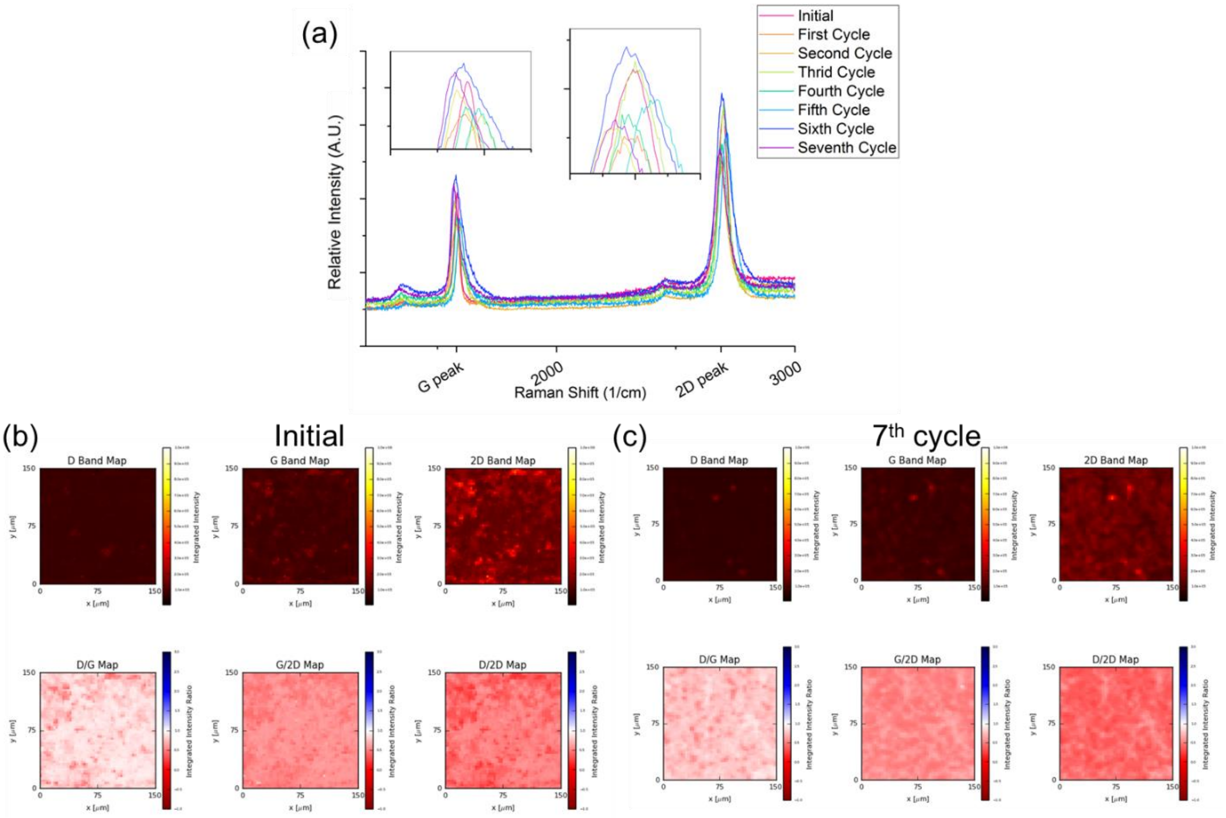

Figure S3. (**a**) Overlaid Raman spectra of graphene surface after several repetitions of ZnO growth/etching processing. (**b**) Raman mappings of D, G and 2D bands and their corresponding ratios after the initial growth and nanowire removal and (**c**) after seventh cycle of growth/removal.

© 2021 by the authors. Licensee MDPI, Basel, Switzerland. This article is an open access article distributed under the terms and conditions of the Creative Commons Attribution (CC BY) license (http://creativecommons.org/licenses/by/4.0/).
